# [2 + 2] Photodimerization of Naphthylvinylpyridines through Cation-π Interactions in Acidic Solution

**DOI:** 10.3390/molecules22030491

**Published:** 2017-03-20

**Authors:** Shinji Yamada, Yuka Nojiri

**Affiliations:** Department of Chemistry, Ochanomizu University, 2-1-1 Otsuka, Bunkyo-ku, Tokyo 112-8610, Japan; yuka.nojiri@konicaminolta.com

**Keywords:** cation-π interaction, [2 + 2] photodimerization, cyclobutane derivative, naphthylvinylpyridine, stereoselective reaction

## Abstract

Irradiation of (*E*)-4-(2-(2-naphthyl)vinyl)pyridine (**1a**) and (*E*)-4-(2-(1-naphthyl)vinyl)pyridine (**1b**) with a 250 W high-pressure mercury lamp in acidic solution afforded *syn*HT dimers in high stereoselectivities. Similar results were obtained by visible light irradiation. On the other hand, when the reactions were carried out under neutral conditions, the stereoselectivities were very low, and the yields were decreased by visible light irradiation. Comparison of the UV-vis spectra between the acidic and the neutral conditions elucidated that the red shift was observed in acidic solutions. These results show that HCl plays essential roles not only in the preorientation of substrates through cation-π interactions, but also in the changes in the absorption properties of substrates that enable visible light reactions.

## 1. Introduction

Controlling the association mode of substrate alkenes during [2 + 2] photodimerization is a key subject in organic photochemistry [1,2,3,4], as it is essential for the regio- and stereoselective formation of cyclobutane dimers. Various approaches for the alignment of molecules have been developed using a variety of organized media [5], organic crystals [6,7,8,9,10], metal–organic frameworks [11,12], metal complexes [13,14], and supramolecular systems [15,16]. Previously, we reported that a cation-π interaction [17] between a pyridinium salt and a benzene ring plays an essential role on the product distribution in the [2 + 2] photodimerization of styrylpyridines [18,19,20]. This preorientation process is responsible for various stereoselective photodimerization reactions not only in solution, but also in crystals [21,22].

**Figure 1:**
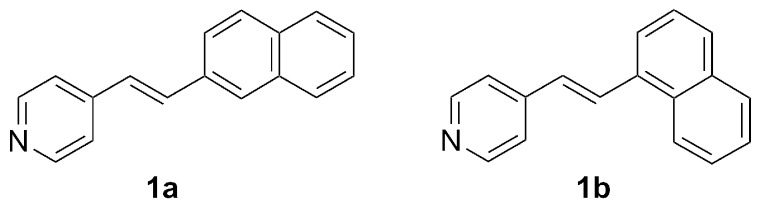
Substrates for photodimerization.

It has been reported that the size of the π-system is significantly correlated with the strength of the cation-π interactions; for example, the interaction energy of a sodium cation with naphthalene is 2–3.5 kcal/mol larger than that with benzene [23,24]. This prompted us to investigate the [2 + 2] photodimerization of naphthylvinylpyridines having more extended π-systems than those of styrylpyridines. In this paper, we report that the photodimerization of naphthylvinylpyridines **1a** and **1b** (Figure 1) in acidic solution provides *syn*HT dimers with very high regio- and stereoselectivities through cation-π interaction, even under irradiation with visible light.

## 2. Results and Discussion

(*E*)-4-(2-(2-Naphthyl)vinyl)pyridine (**1a**) [25] and (*E*)-4-(2-(1-naphthyl)vinyl)pyridine (**1b**) [25] were employed as substrates for the photochemical reactions, the π-conjugate systems of which differed from each other (Figure 1). Irradiation of **1a** in a 1.0 M THF solution with a 450 W high-pressure mercury lamp for 8 h under neutral conditions afforded all four possible dimers **2a**–**5a**. *Syn*HT dimer **3a** was the major product, but the selectivity was quite low—as shown in Table 1 (entry 1). The conversion and the product ratios were determined based on ^1^H-NMR spectra. Though the reaction was carried out in a 0.067 M MeOH solution due to the much lower solubility of **1a** than that in THF, a similar result was observed (entry 2). Photolysis in the presence of 1 equiv. of conc. hydrochloric acid resulted in remarkable changes in the product distribution (entry 3); the *syn*HT adduct **2a** increased to become the major product along with a small amount of **3a** and **4a**. As the amount of HCl loading increased to 3 equiv., the yield of the *syn*HT dimer **2a** increased to 93% (entry 4).

**Figure 2:**
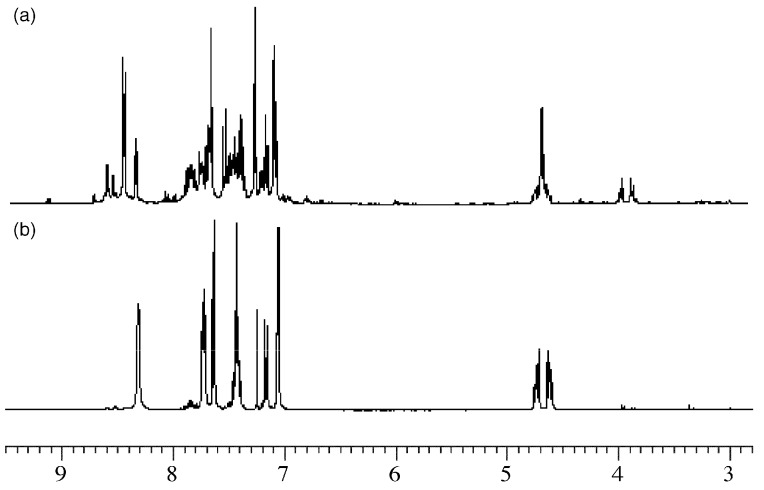
^1^H-NMR spectra in CDCl_3_ for (**a**) **1a** after irradiation for 24 h; and (**b**) **1a** after irradiation in the presence of 3 eq. of HCl and successive neutralization.

Figure 2a,b show the ^1^H-NMR spectra of **1a** after irradiation in the absence and presence of 3 equiv. of HCl, respectively. A comparison clearly demonstrates the significant effect of the acid on the product distribution. Figure 2a shows multi-peaks around δ 3.7 to 4.8 for the methine protons of the four cyclobutane dimers. On the other hand, in the presence of 3 equiv. of HCl, the four methine protons appear at δ 4.59 and 4.62, indicating exclusive formation of **2a** (Figure 2b).

Irradiation of **1b** in the absence of the acid gave a mixture of **2b**–**4b** and (*Z*)-isomer (entry 5). In the presence of 1 equiv. of HCl, the solubility of **1b** decreased; therefore, the reaction was carried out in a 0.67 M MeOH solution. The acid demonstrated a significant effect similar to that in the case of **1a**; *syn*HT dimer **2b** was obtained in 75% yield with no other dimers along with 25% of (*Z*)-isomer (entry 6). A survey of the solvents found that a 9:1 mixture of MeOH and H_2_O had a good solubility for **1b**·HCl. Using this solvent system, irradiation of a 1.0 M solution of **1b** in the presence of acid significantly improved the selectivity to 94%, suggesting the importance of concentration in obtaining good selectivities in these reactions (entries 7 and 8).

Another remarkable feature observed under the acidic conditions was that visible light could also induce photodimerization. When the irradiation of **1a** was carried out with a 250 W high-pressure mercury lamp through a UV-cut filter for 3 h, only (*Z*)-isomer was produced in 7% yield (Table 2, entry 1). On the contrary, in the presence of HCl, the reaction proceeded to provide *syn*HT dimer **2a** in a similar selectivity to that obtained by UV-light irradiation (entry 2). Irradiation of **1b** in the absence of the acid gave dimers **2b** and **3b** in low yields, whereas in the presence of the acid, **2b** was produced in a significantly high yield (entries 3 and 4).

**Figure 3:**
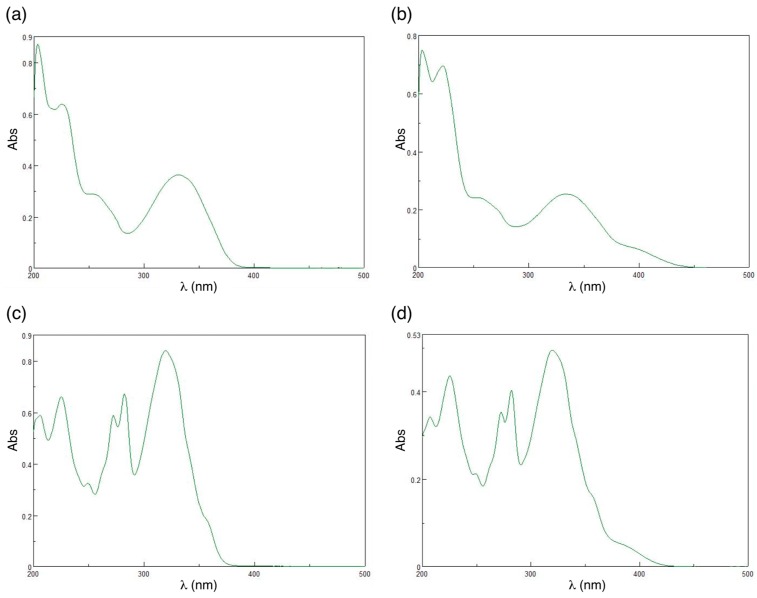
UV-vis spectra in EtOH for (**a**) **1a** (2.09 × 10^−5^ mol/L); (**b**) **1a**·HCl (1.80 × 10^−5^ mol/L); (**c**) **1b** (2.34 × 10^−5^ mol/L); and (**d**) **1b**·HCl (1.82 × 10^−5^ mol/L).

The remarkable differences in the photoreactivities whether in the presence or absence of HCl can be explained by differences in their absorption properties. UV-vis spectra for **1a**, **1b**, **1a**·HCl, and **1b**·HCl are shown in Figure 3. The longest absorption bands of **1a** and **1b** in MeOH appeared at 319.0 and 331.0 nm, respectively. On the other hand, in the presence of HCl these bands were shifted to longer wavelengths and observed at 331.0 and ca. 360 nm, respectively, the shoulders of which exceeded 400 nm. These changes in the absorption properties induced by the acid are thought to be responsible for the visible light photodimerization in acidic media.

**Figure 4:**
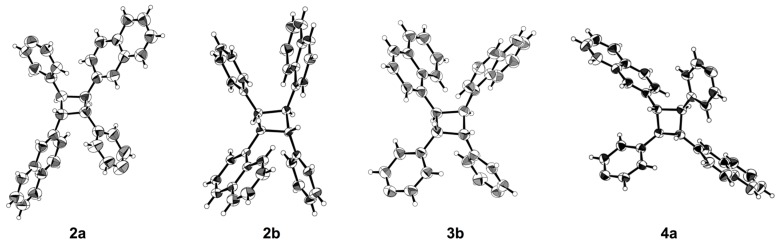
X-ray structures of **2a**, **2b**·2HCl, **3b**, and **4a**.

The structures of the product dimers were determined from X-ray crystallographic analyses and the ^1^H-NMR and MS spectra. The X-ray analyses were carried out for **2a**, **2b**·2HCl, **3b,** and **4a**, as single crystals suitable for X-ray analyses were obtained. Figure 4 clearly shows that **2a** and **2b** are *syn*HT dimers, and **3b** and **4a** are *syn*HH and *anti*HT dimers, respectively. Based on MS and ^1^H-NMR analyses, **3a** and **5a** were assigned as *syn*HH and *anti*HH dimers, respectively. In their MS spectra, the fragment peaks of *m*/*z* 231 and 280 were observed. The peak of *m*/*z* 231 corresponds to a naphthylethylpyridine moiety, and the peak of *m/z* 280 corresponds to a binaphthylethane moiety, indicating their head-to-head structures (Figures S8, S9, S12, and S13). The methine protons at δ 4.67 and 4.69 for **3a** and δ 3.88 and 3.97 for **5a** are very close to those of related *syn*HH and *anti*HH dimers, respectively [18]. The assignment of **4b** was also performed by MS spectra; the observations of a base peak of *m*/*z* 231 with no peak of *m*/*z* 280 are characteristic of the head-to-tail structures (Figures S10 and S11). In addition, since **2b** was assigned as a *syn*HT dimer by X-ray analysis, **4b** should be an *anti*HT dimer.

**Figure 5:**
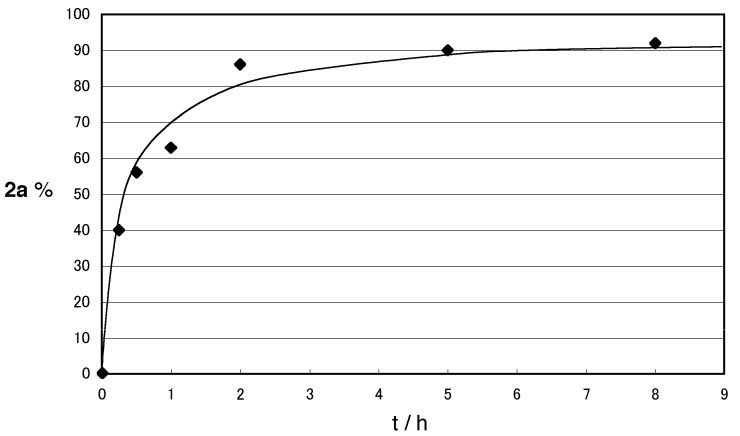
Plots of the yield of **2a** vs. irradiation time.

To obtain mechanistic insights into the dimerization process, the dependence of product distribution on the irradiation time was investigated. Figure 5 shows a plots of the yields of *syn*HT dimer **2a** vs. irradiation time. The yield of **2a** increased with increased irradiation time, with the curve following second-order kinetics. This suggests that the dimerization process does not involve any other processes, such as a reverse reaction or isomerization reaction. The second-order kinetics was confirmed by plots of t vs. 1/[**1a**], in which a linear correlation was observed (Figure S14).

**Scheme 1:**
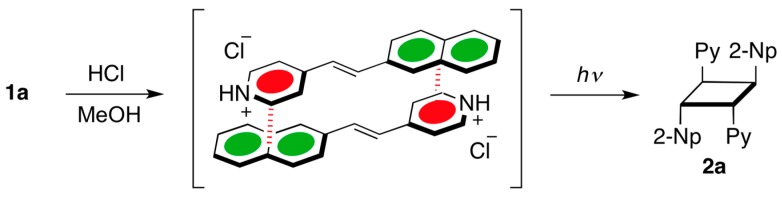
Formation of a *syn*HT dimer **2a** through cation-π interactions.

The preference for the formation of the *syn*HT dimer in the presence of HCl agrees with that in the photodimerization of styrylpyridine [18]. This *syn*HT preference indicates that the intermediate naphthylvinylpyridinium salts form preorganized head-to-tail molecular dimers through intermolecular cation-π interactions, as shown in Scheme 1. The fact that the selectivities observed in the photodimerization of **1a** and **1b** are much higher than that in the case of styrylpyridine (71% selectivity) [18] suggests a significant effect of the naphthyl group on the intermolecular cation-π interactions, regardless of the differences in the π-conjugate systems.

It has been reported that the larger aromatic π-conjugate systems are more effective for cation-π interactions. For example, the threshold collision-induced dissociation technique clarified that extended π-network leads to an increase in the strength of the cation-π interaction due to increased polarizability of the ligand [23]. The B3LYP calculations for the binding energy of Na^+^···naphthalene predicted that the energy is larger than that of Na^+^···benzene [24]. These experimental and theoretical studies agree with the higher selectivities observed in the photodimerization of **1a** and **1b** than that of styrylpyridine. 

## 3. Materials and Methods

### 3.1. General Methods

Melting points were determined with a Yanaco model MP microscope (Yanaco, Tokyo, Japan). Column chromatography was carried out using silica gel 60 N (Kanto Chemical, Tokyo, Japan). Thin layer chromatography (TLC) was carried out on a Merck silica gel 60 PF_254_ (Merck, Tokyo, Japan). IR spectra were obtained on FT/IR-410 spectrometer (JASCO, Tokyo, Japan) as neat films between NaCl plates, or KBr pellets. NMR spectra were recorded on JEOL EX-400 spectrometer (JEOL, Tokyo, Japan). ^1^H-NMR spectra were obtained at 400 MHz as dilute solution in CDCl_3_, and the chemical shifts were reported relative to internal TMS. Low-resolution mass spectra were recorded at ionizing voltage of 70 eV by electron impact. High-resolution mass spectra were recorded on Exactive Orbitrap LC-MS (Thermo-Fischer, Yokohama, Japan). UV/vis spectra were recorded on JASCO V-650DS spectrometer (JASCO, Tokyo, Japan) in MeOH. UV irradiation was performed using a 450 W high-pressure mercury lamp (USHIO UM453, Tokyo, Japan). Visible-light irradiation was performed using a 250 W high-pressure mercury lamp (ASAHI SPECTRA REX-250, Tokyo, Japan) through quartz fibers with a UV-cut filter.

### 3.2. Representative Procedure for Irradiation of Naphthylvinylpyridines under Acidic Conditions

A solution of naphthylvinylpyridine (0.1 mmol) in 100 μL of MeOH containing conc. HCl was irradiated for 8 h through a Pyrex filter with a 450 W high-pressure mercury lamp. The solution was neutralized with sat. NaHCO_3_, and the solvent was evaporated to give an oil. This oil was extracted with CH_2_Cl_2_ three times, and the combined organic layer was dried over anhydrous MgSO_4_. Evaporation of the solvent gave a crude product, which was subjected to column chromatography on silica gel with a 7:2:1 mixture of ethyl acetate, hexane, and methanol as an eluent. The product ratio was determined by the ^1^H-NMR spectrum of the crude product. Visible-light irradiation of the above-mentioned solution was carried out with a 250 W high-pressure mercury lamp for 3 h through a UV-cut filter. The work-up followed the same procedure as that described above.

Compound **2a**: Pale-yellow crystals; m.p. 196–197 °C; ^1^H-NMR (400 MHz, CDCl_3_) δ 8.32 (d, *J* = 5.6 Hz, 4H), 7.72–7.75 (m, 4H), 7.63–7.65 (m, 4H), 7.42–7.45 (m, 4H), 7.17 (dd, *J* = 8.6, 2.0 Hz, 2H), 7.06 (d, *J* = 6.0 Hz, 4H), 4.72–4.77 (m, 2H), 4.60–4.64 (m, 2H); IR (KBr) 3018, 1597, 1552, 1509, 1414, 994, 861, 813, 763 cm^−1^; MS *m*/*z* 462 (M^+^, 0.6%), 232 (20), 231 (100), 230 (64), 203 (8), 202 (13). HRMS calcd. for C_34_H_27_N_2_ 463.21742, (M + H)^+^, found 463.21501.

Compound **3a**: Pale-yellow crystals; m.p. 113–114 °C; ^1^H-NMR (400 MHz, CDCl_3_) δ 8.43 (d, *J* = 4.4 Hz, 4H), 7.65–7.69 (m, 6H), 7.53 (d, *J* = 8.0 Hz, 2H), 7.34–7.42 (m, 4H), 7.15 (dd, *J* = 8.6, 2.0 Hz, 2H), 7.09 (d, *J* = 6.0 Hz, 4H), 4.66–4.70 (m, 4H); IR (KBr) 3053, 2972, 1599, 1508, 1422, 1115, 995, 820, 746 cm^−1^; MS *m*/*z* 462 (M^+^, 0.6%), 280 (26), 279 (18), 232 (39), 231 (100), 230 (96), 202 (13). HRMS calcd. for C_34_H_27_N_2_ 463.21742 (M + H)^+^, found 463.21715.

Compound **4a**: Pale-yellow crystals; m.p. 225–226 °C; ^1^H–NMR (400 MHz, CDCl_3_) δ 8.53 (d, *J* = 4.8 Hz, 4H), 7.80–7.89 (m, 8H), 7.46–7.52 (m, 6H), 7.21 (d, *J* = 6.0 Hz, 4H), 3.96–4.01 (m, 2H), 3.84–3.89 (m, 2H); IR (KBr) 3053, 2927, 1597, 1415, 1068, 817, 751 cm^−1^; MS *m*/*z* 462 (M^+^, 1.2%), 232 (26), 231 (100), 230 (89), 202 (18). HRMS calcd. for C_34_H_27_N_2_ 463.21742 (M + H)^+^, found 463.21560.

Compound **5a**: A colorless oil; ^1^H-NMR (400 MHz, CDCl_3_) δ 8.57 (d, *J* = 6.0 Hz, 4H), 7.78–7.83 (m, 6H), 7.75 (s, 2H), 7.45–7.48 (m, 4H), 7.40 (dd, *J* = 8.6, 2.0 Hz, 2H), 7.25–7.26 (m, 4H), 3.96−3.98 (m, 2H), 3.86–3.89 (m, 2H); IR (KBr) 3054, 2927, 1597, 1415, 1068, 817, 751 cm^−1^; MS *m*/*z* 462 (M^+^, 3.7%), 370 (13), 281 (11), 280 (44), 279 (27), 278 (15), 232 (37), 231 (100), 230 (89), 229 (13), 202 (15). HRMS calcd. for C_34_H_27_N_2_ 463.21742 (M + H)^+^, found 463.21693.

Compound **2b**: Pale-yellow crystals; 263–265 °C; ^1^H-NMR (400 MHz, CDCl_3_) δ 8.20 (d, *J* = 6.0 Hz, 4H), 7.97 (d, *J* = 8.4 Hz, 2H), 7.79 (d, *J* = 8.0 Hz, 2H), 7.68 (d, *J* = 8.4 Hz, 2H), 7.43–7.50 (m, 6H), 7.36–7.40 (m, 2H), 7.02 (dd, *J* = 4.4, 1.2 Hz, 4H), 5.26 (dd, *J* = 9.6, 7.6 Hz, 2H), 4.86 (dd, *J* = 9.6, 7.6 Hz, 2H); IR (KBr) 3035, 1597, 1507, 1478, 1416, 996, 801, 773, 681 cm^−1^; MS *m/z* 462 (M^+^, 0.2%), 232 (30), 231 (100), 230 (81), 203 (7), 202 (12), 153 (21), 152 (13). HRMS calcd. for C_34_H_27_N_2_ 463.21742 (M + H)^+^, found 463.21440.

Compound **3b**: White crystals; 206–208 °C; ^1^H-NMR (400 MHz, CDCl_3_) δ 8.45 (d, *J* = 6.0 Hz, 4H), 7.89 (d, *J* = 8.4 Hz, 2H), 7.72 (d, *J* = 8.4 Hz, 2H), 7.60 (d, *J* = 8.4 Hz, 2H), 7.28–7.38 (m, 6H), 7.19–7.23 (m, 2H), 7.12 (d, *J* = 6.0 Hz, 4H), 5.40–5.42 (m, 2H), 4.61–4.63 (m, 2H); IR (KBr) 3050, 1941, 1601, 1548, 1509, 1408, 999, 784 cm^−1^; MS *m*/*z* 462 (M^+^, 1.1%), 280 (10), 279 (7), 232 (27), 231 (100), 230 (62), 202 (10), 153 (15), 152 (11). HRMS calcd. for C_34_H_27_N_2_ 463.21742 (M + H)^+^, found 463.21465.

Compound **4b**: A pale-yellow oil: ^1^H-NMR (400 MHz, CDCl_3_) δ 8.48 (d, *J* = 5.6 Hz, 4H), 7.86 (d, *J* = 8.4 Hz, 2H), 7.82 (d, *J* = 6.4 Hz, 4H), 7.65 (d, *J* = 8.4 Hz, 2H), 7.58 (t, *J* = 7.2 Hz, 2H), 7.44 (t, *J* = 7.2 Hz, 2H), 7.22–7.25 (m, 2H), 7.21 (d, *J* = 6.4 Hz, 4H), 4.60–4.65 (m, 2H), 4.07–4.12 (m, 2H); IR (KBr) 3049, 2915, 1598, 1559, 1507, 1413, 993, 800, 781 cm^−1^; MS *m*/*z* 462 (M^+^, 1.9%), 232 (47), 231 (100), 230 (89), 202 (12). HRMS calcd. for C_34_H_27_N_2_ 463.21742 (M + H)^+^, found 463.21515.

### 3.3. X-ray Crystallography

Single crystals of dimers were mounted on a CryoLoop (Hampton Research, Aliso Viejo, CA, USA). All measurements were made on a Rigaku RAXIS RAPID II imaging plate area detector (Rigaku, Tokyo, Japan) with graphite monochromated Cu-Kα radiation at −150 °C. The structures were solved by the direct method and refined by full-matrix least squares on *F*^2^ using SHELXL 97 [26]. Crystal Data for **2a** (*M* = 462.59 g/mol): monoclinic, space group *P*2_1_, *a* = 10.5616(6) Å, *b* = 9.5453(6) Å, *c* = 13.5147(7) Å, β = 110.173(2)°, *V* = 1278.89(12) Å^3^, *Z* = 4, *D*_calc_ = 1.201 g/cm^3^, 14,679 reflections measured, 4523 unique (*R*_int_ = 0.0404). The final *R*1 was 0.0761 (*I* > 2σ(*I*)) and *wR*2 was 0.2912 (all data). CCDC 747473 contains the supplementary crystallographic data for this paper. Crystal Data for **2b**·2HCl·2H_2_O (*M* = 571.54 g/mol): monoclinic, space group *Cc*, *a* = 13.6293(5) Å, *b* = 17.9154(7) Å, *c* = 12.9243(5) Å, β = 110.971(2)°, *V* = 2946.8(2) Å^3^, *Z* = 4, *D*_calc_ = 1.288 g/cm^3^, 16,003 reflections measured, 4988 unique (*R*_int_ = 0.0583). The final *R*1 was 0.0552 (*I* > 2σ(*I*)) and *wR*2 was 0.1779 (all data). CCDC 747474 contains the supplementary crystallographic data for this paper. Crystal Data for **3b** (*M* = 462.59 g/mol): monoclinic, space group *P*2_1_/*c*, *a* = 13.4512(6) Å, *b* = 13.1979(5) Å, *c* = 14.7875(7) Å, β = 110.782(2)°, *V* = 2454.38(19) Å^3^, *Z* = 4, *D*_calc_ = 1.252 g/cm^3^, 26,569 reflections measured, 4443 unique (*R*_int_ = 0.0451). The final *R*1 was 0.0570 (*I* > 2σ(*I*)) and *wR*2 was 0.2043 (all data). CCDC 747475 contains the supplementary crystallographic data for this paper. Crystal Data for **4a** (*M* = 462.59 g/mol): orthorhombic, space group *Pbca*, *a* = 22.5434(4) Å, *b* = 8.91677(10) Å, *c* = 49.7495(10) Å, *V* = 10,000.4(3) Å^3^, *Z* = 12, *D*_calc_ = 0.922 g/cm^3^, 104,123 reflections measured, 9132 unique (*R*_int_ = 0.0690). The final *R*1 was 0.0648 (*I* > 2σ(*I*)) and *wR*2 was 0.2166 (all data). CCDC 747476 contains the supplementary crystallographic data for this paper. These data can be obtained free of charge via http://www.ccdc.cam.ac.uk/conts/retrieving.html (or from the CCDC, 12 Union Road, Cambridge CB2 1EZ, UK; Fax: +44 1223 336033; E-mail: deposit@ccdc.cam.ac.uk).

## 4. Conclusions

[2 + 2] Photodimerization of naphthylvinylpyridines in the presence of HCl in MeOH gave *syn*HT dimers with excellent regio- and stereoselectivities. This *syn*HT preference strongly suggests the formation of preorganized head-to-tail molecular dimers from the intermediate naphthylvinylpyridinium salts through intermolecular cation-π interactions. The much higher selectivities compared to that of styrylpyridine reveal the effectiveness of the extended π-systems on the selectivity of the photodimerization. Another remarkable feature observed under the acidic conditions was that the visible light was also capable of inducing [2 + 2] photocycloaddition. These results show that HCl plays essential roles in both the cation-π complex formation and the changes in the absorption properties of substrates that enable visible light reactions. As longer conjugate systems generally form a stronger cation-π complex with a cation, such systems are useful not only in [2 + 2] photocycloaddition, but also in various cation-π-controlled organic reactions.

## Figures and Tables

**Table 1:**
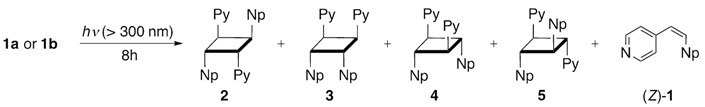
UV irradiation of **1a** and **1b**. ^a^

Entry	Compd.	HCl (eq.)	Solvent ^b^	Conv. (%) ^c^	Products (%)				
					2	3	4	5	(*Z*)-1
1	**1a** ^d^	0	THF	99	21	46	14	17	0
2	**1a**	0	MeOH ^e^	82	22	50	12	4	11
3	**1a**	1	MeOH	98	90	6	4	0	0
4	**1a**	3	MeOH	99	93	2	3	2	0
5	**1b**	0	MeOH	91	45	35	6	0	14
6	**1b**	1	MeOH ^f^	91	75	0	0	0	25
7	**1b**	1	MeOH/H_2_O	93	94	0	0	0	6
8	**1b**	3	MeOH/H_2_O	93	94	0	0	0	6

^a^ External irradiation was carried out through Pyrex filter in cooling water. ^b^ A 1.0 M solution was employed unless otherwise noted. ^c^ Determined by ^1^H-NMR. ^d^ Naphtha [2,1-*h*] isoquinoline was obtained in 2% yield. ^e^ A 0.067 M solution was used. ^f^ A 0.67 M solution was used.

**Table 2:**
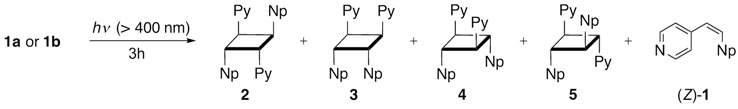
Visible-light irradiation of **1a** and **1b**. ^a^

Entry	Compd.	HCl (eq.)	Solvent	Conv. (%)	Products (%) ^b,c^				
					2	3	4	5	(*Z*)-1
1	**1a**	0	THF	7	0	0	0	0	100
2	**1a**	1	MeOH	99	93	3	2	2	0
3	**1b**	0	MeOH	48	46	39	0	0	15
4	**1b**	1	MeOH/H_2_O	88	90	0	0	0	10

^a^ Spot irradiation was carried out through quartz fibers with a UV-cut filter. ^b^ A 1.0 M solution was employed unless otherwise noted. ^c^ Determined by ^1^H-NMR.

## Supplementary Materials

Supplementary materials are available online. Figures S1–S7: ^1^H-NMR spectra for **2a**–**5a** and **2b**–**4b**, Figures S8–S13: MS spectra for **3a**, **4b** and **5a**, Figure S14: Plots of 1/[1a] vs t.
