# Supplementary material for: [2 + 2] Photodimerization of Naphthylvinylpyridines through Cation-π Interactions in Acidic Solution
Source: Molecules. 2017 Mar 20;22(3):491. doi: 10.3390/molecules22030491 (PMC6155432; doi:10.3390/molecules22030491)
Supplement: Supplementary file 1 [file molecules-22-00491-s001.pdf]

Supplementary Materials

[2+2] Photodimerization of Naphthylvinylpyridines through Cation-π Interactions in Acidic Solution

Shinji Yamada* and Yuka Nojiri

Department of Chemistry, Ochanomizu University, 2-1-1 Otsuka, Bunkyo-ku, Tokyo 112-8610 (Japan)

Fax: (+81) 3-5978-5349; E-mail: yamada.shinji@ocha.ac.jp

Table of Contents

1. ^{1}H NMR spectra for **2a**-**5a** and **2b**-**4b** S1-S8

2. MS spectra for **3a**, **4b** and **5a** S9-S11

3. Plots of second-order kinetics for the formation of **2a** S12

1. ^{1}H NMR spectra for 2a-5a and 2b-4b

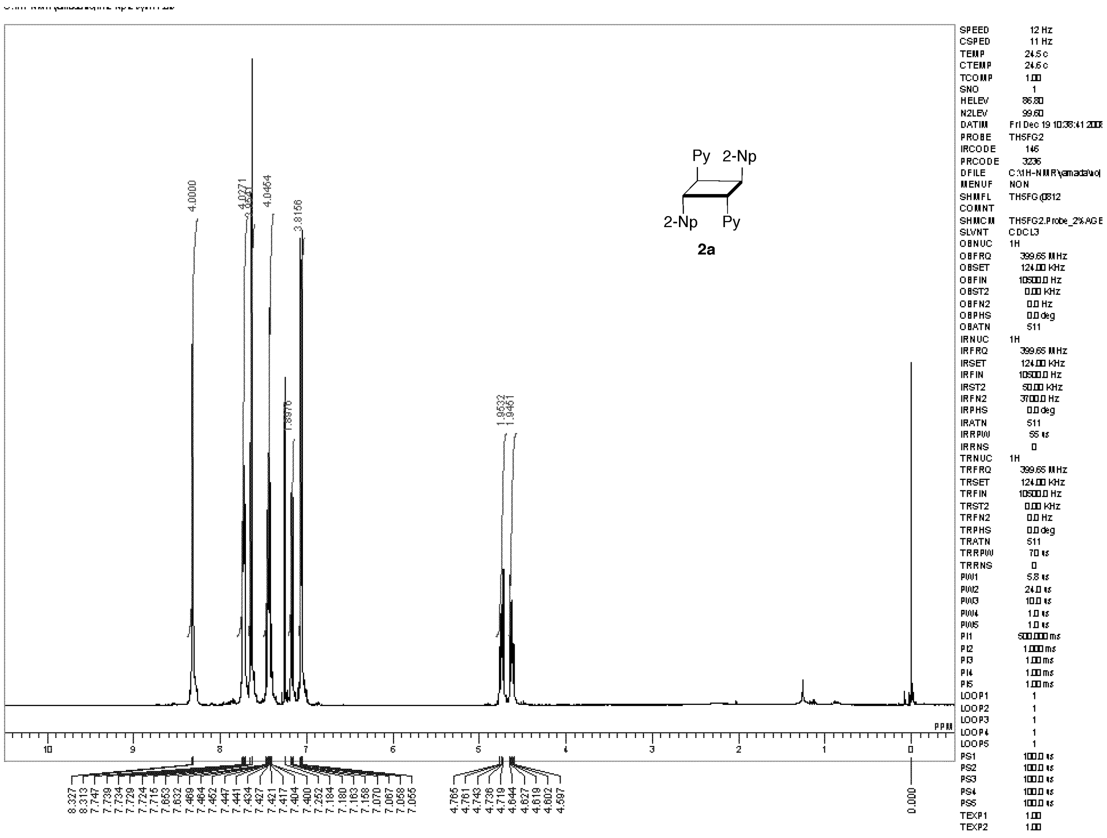

Figure S1. ^{1}H NMR spectrum for **2a**

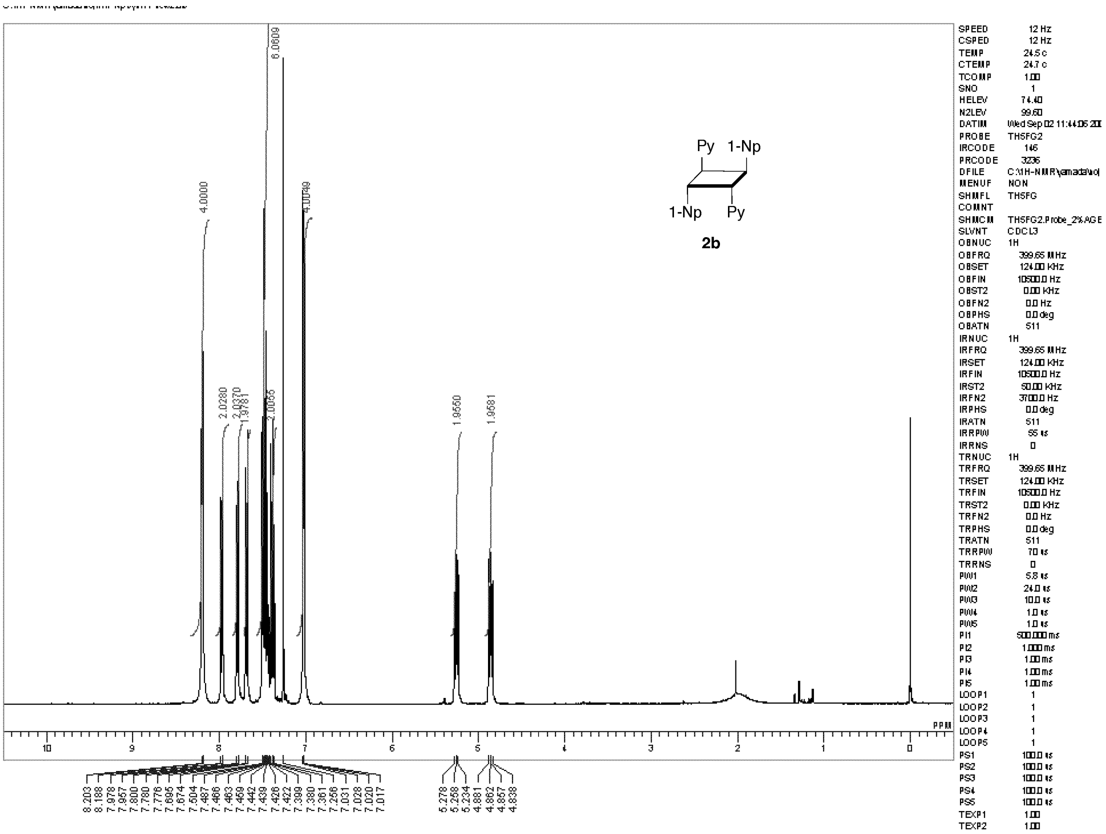

Figure S2. ^{1}H NMR spectrum for **2b**

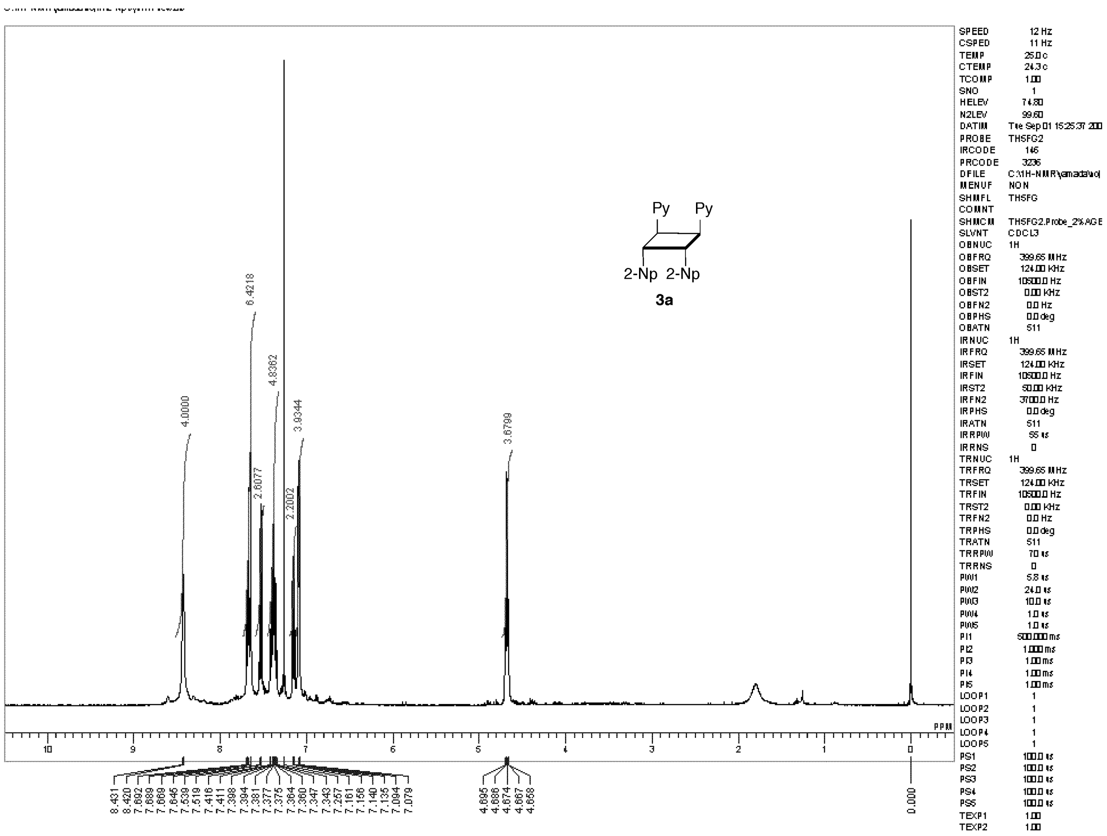

Figure S3. ^{1}H NMR spectrum for **3a**

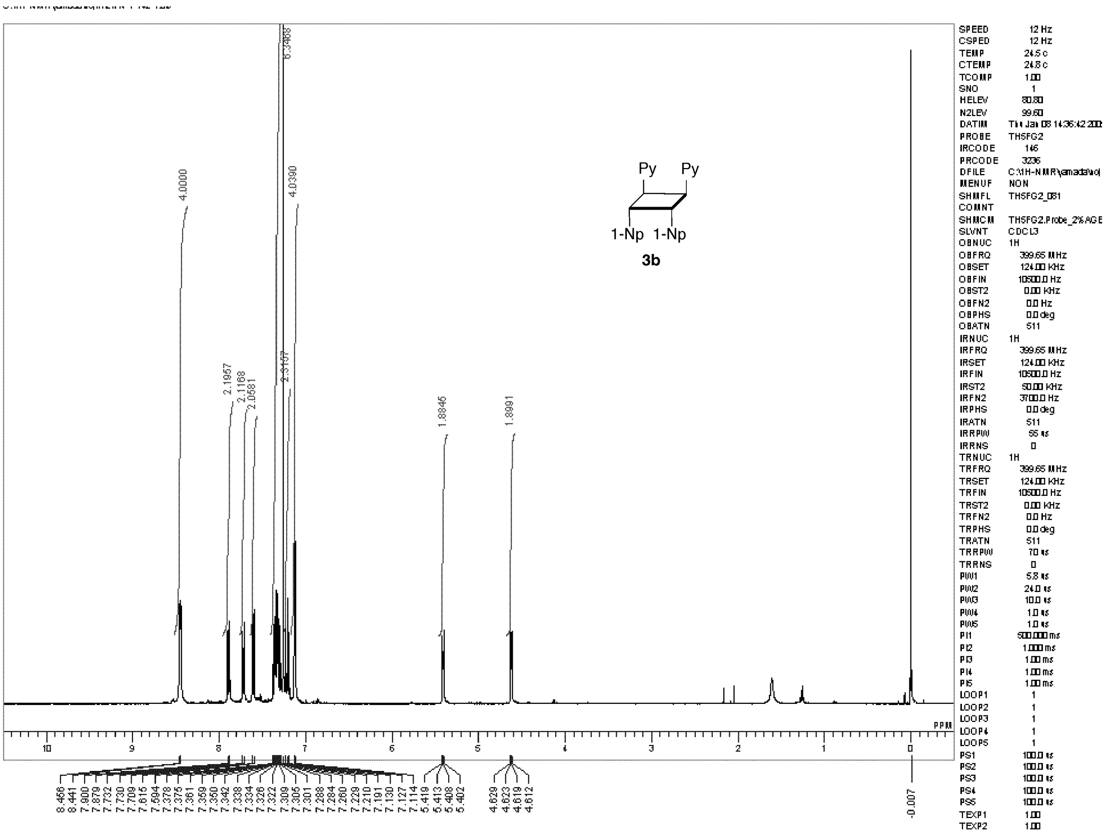

Figure S4. ^{1}H NMR spectrum for **3b**

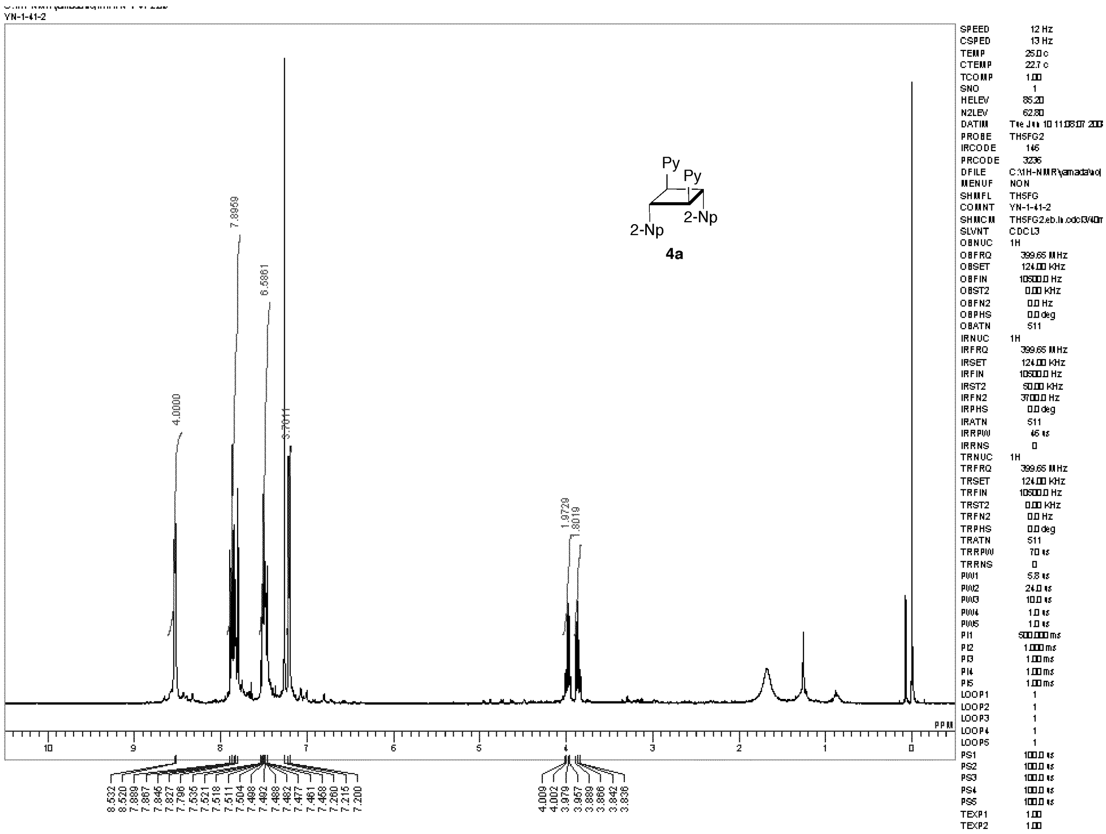

Figure S5. ^{1}H NMR spectrum for **4a**

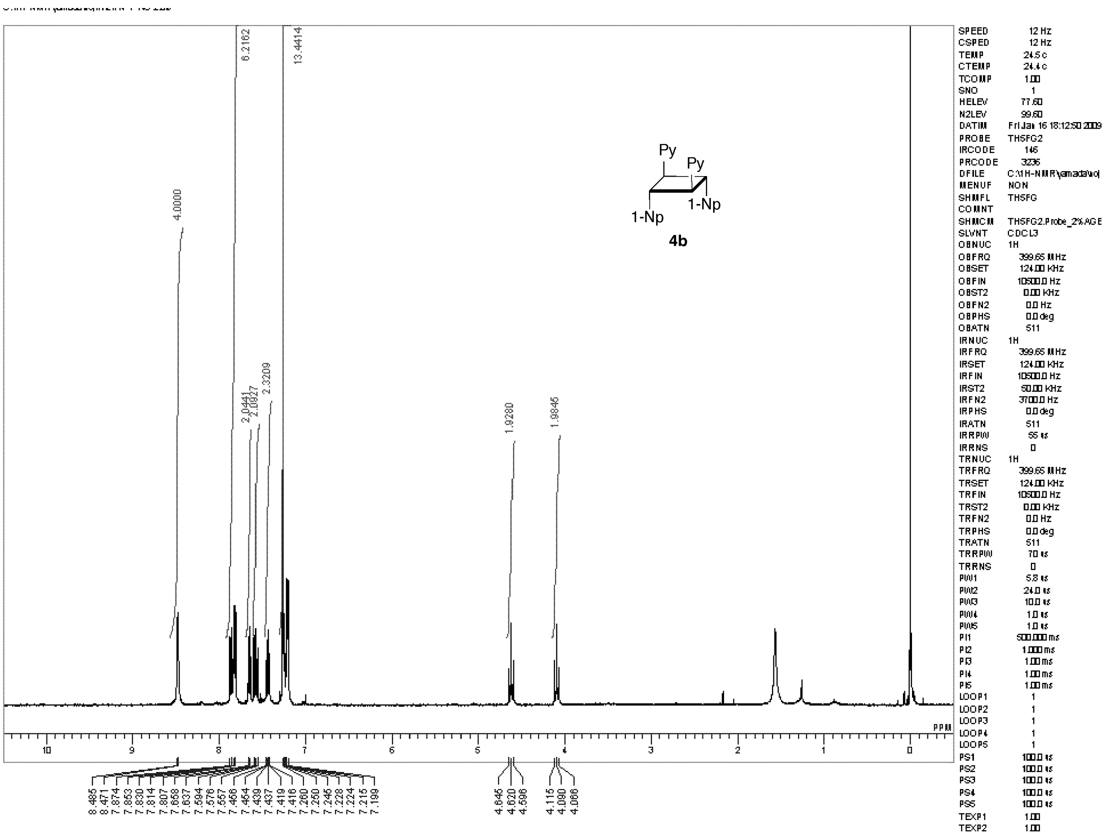

Figure S6. ^{1}H NMR spectrum for **4b**

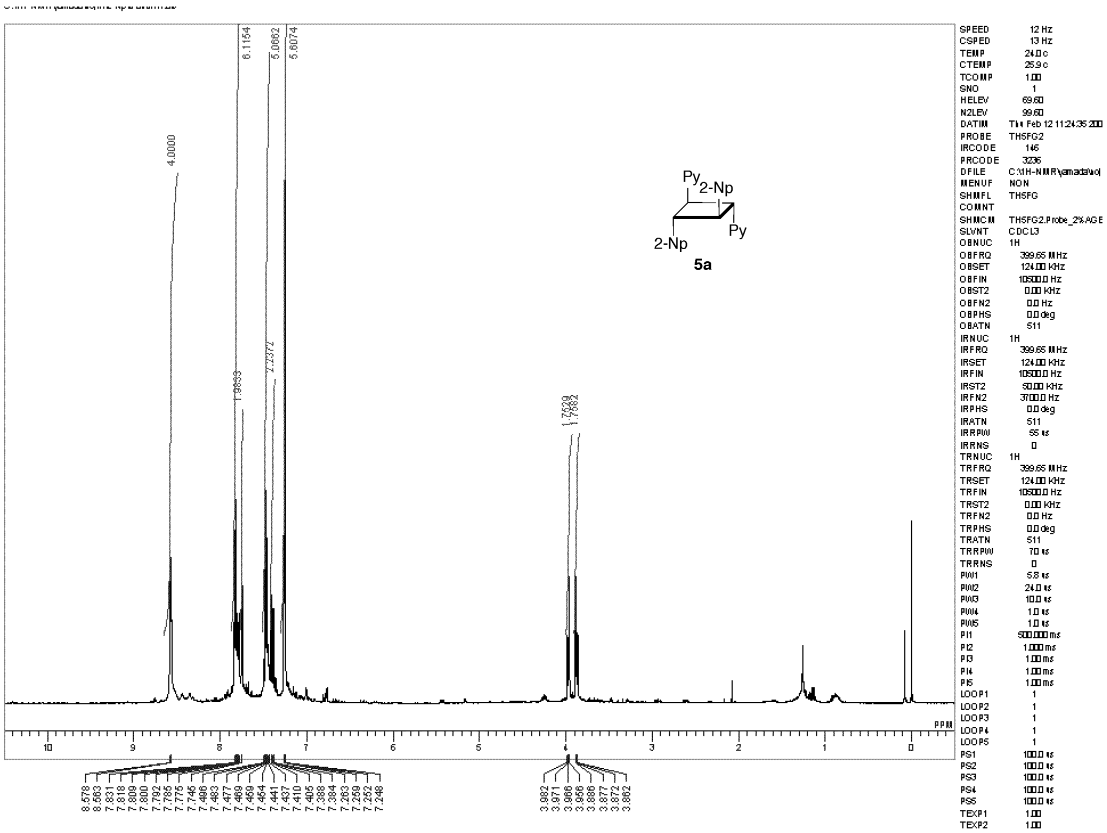

Figure S7. ^{1}H NMR spectrum for **5a**

2. MS spectra for 3a, 4b and 5a

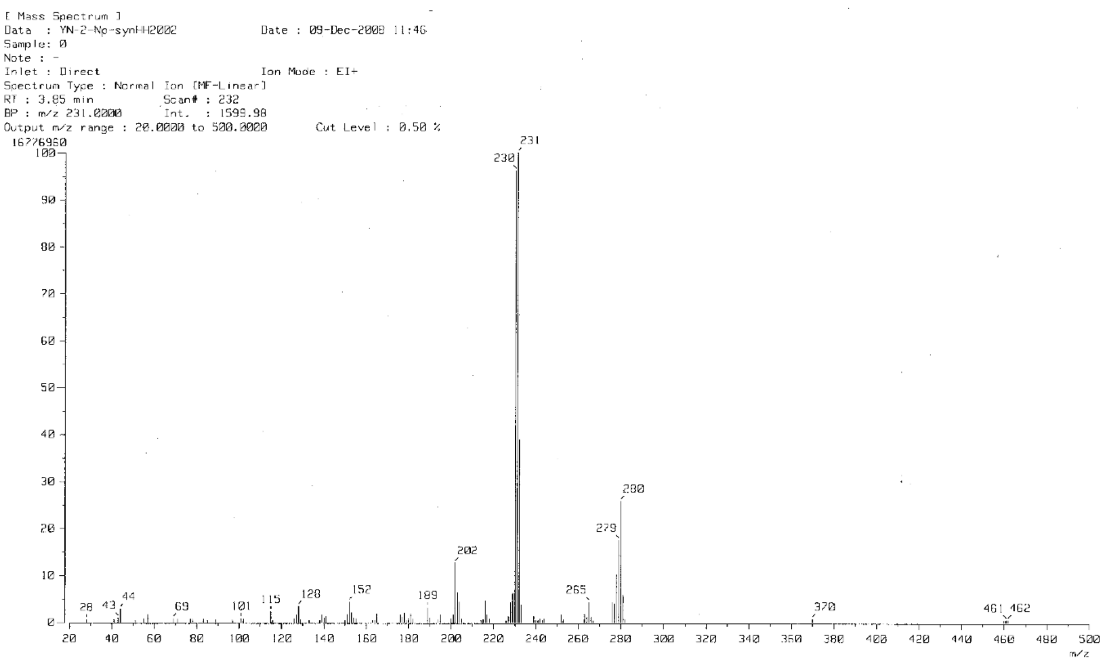

Figure S8. MS spectrum of 3a

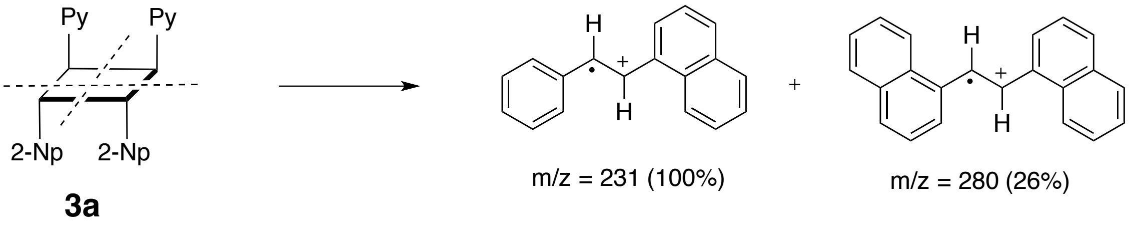

Figure S9. Determination of a head-to-head structure of **3a**

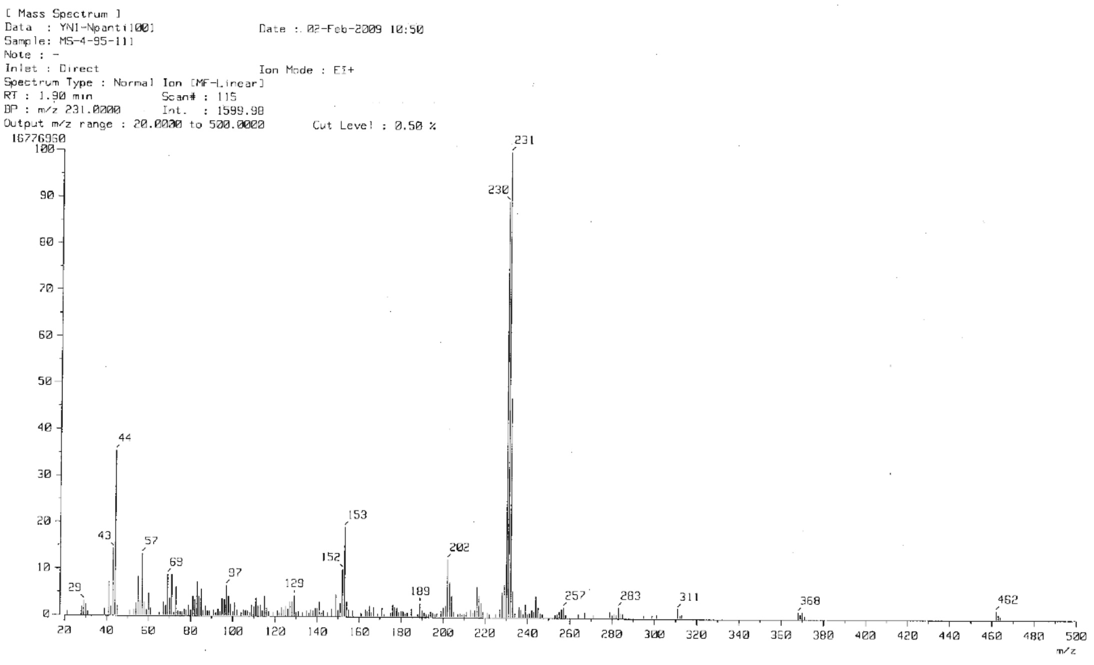

Figure S10. MS spectra for **4b**

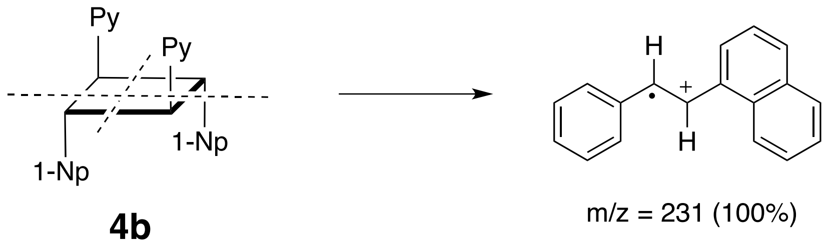

Figure S11. Determination of a head-to-tail structure of **4b**

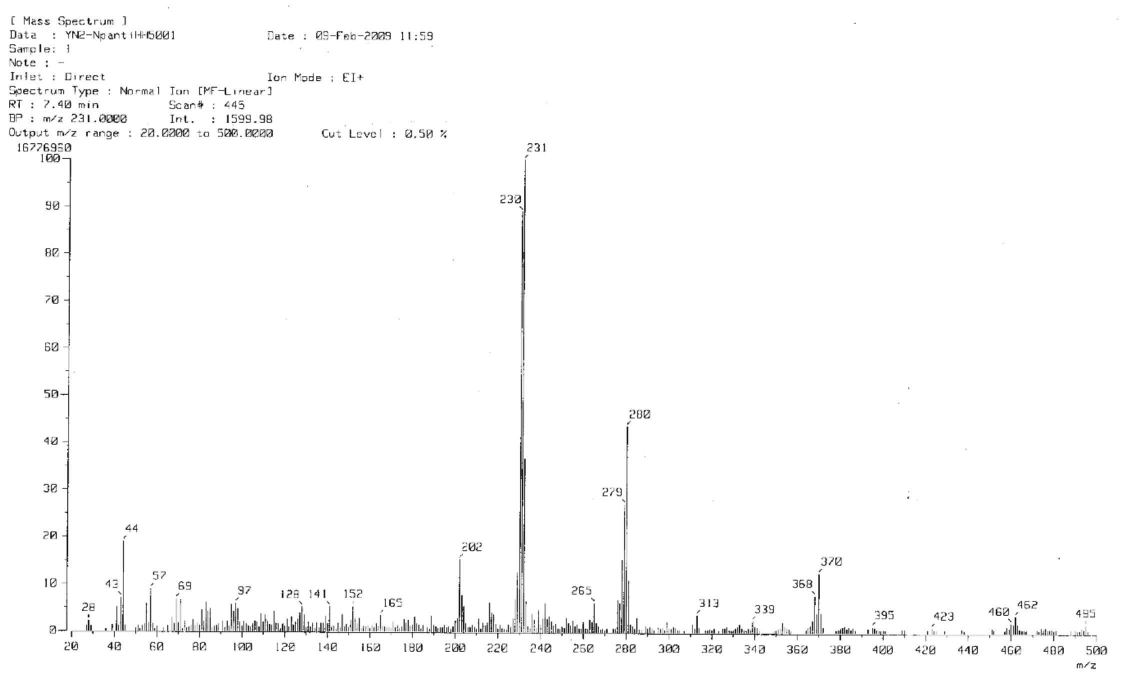

Figure S12. MS spectrum of **5a**

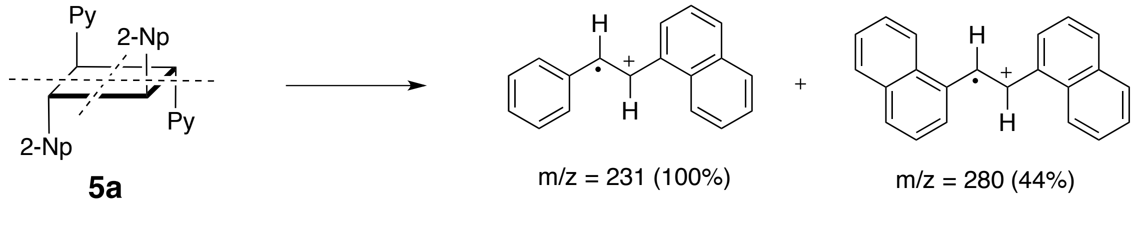

Figure S13. Determination of a head-to-head structure of **5a**

3. Plot of second-order kinetics for the formation of 2a

1a + **1a** → **2a**

$d[\mathbf{1a}]/dt = -k[\mathbf{1a}]^2$

$-d[\mathbf{1a}]/[\mathbf{1a}]^2 = -k dt$

$1/[\mathbf{1a}] - 1/[\mathbf{1a}]_0 = kt$

$1/[\mathbf{1a}] = kt + 1/[\mathbf{1a}]_0 \quad ([\mathbf{1a}] = [\mathbf{1a}]_0/1+kt[\mathbf{1a}]_0)$

Time (h)	[1a] (M)	1/[1a]
0	1	1
0.25	0.59	1.7
0.5	0.43	2.3
1	0.36	2.8
2	0.09	11
5	0.05	20
8	0.02	50

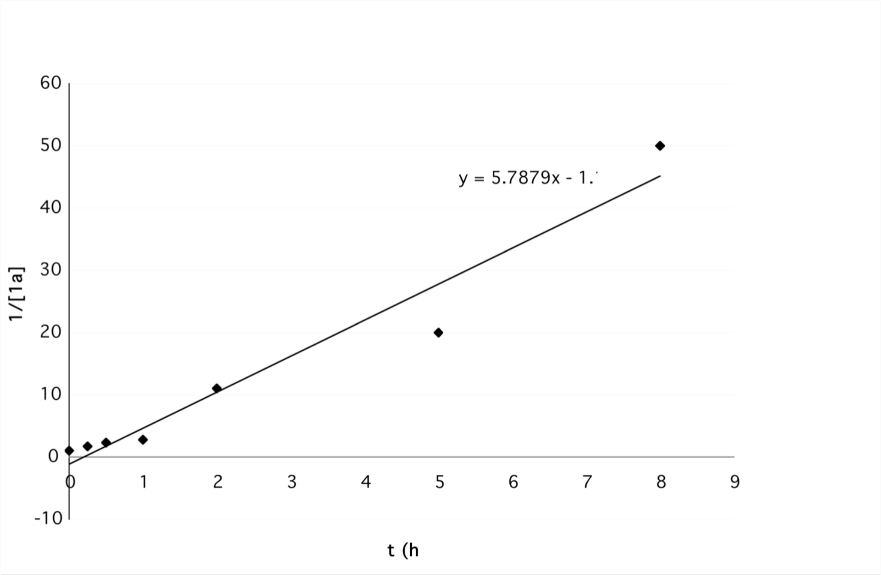

Figure S14. Plot of second-order kinetics for the formation of **2a**
